# Outstanding Reviewers for *Chemical Science* in 2020

**DOI:** 10.1039/d1sc90097h

**Published:** 2021-05-24

## Abstract

We would like to take this opportunity to highlight the Outstanding Reviewers for *Chemical Science* in 2020, as selected by the editorial team for their significant contribution to the journal.
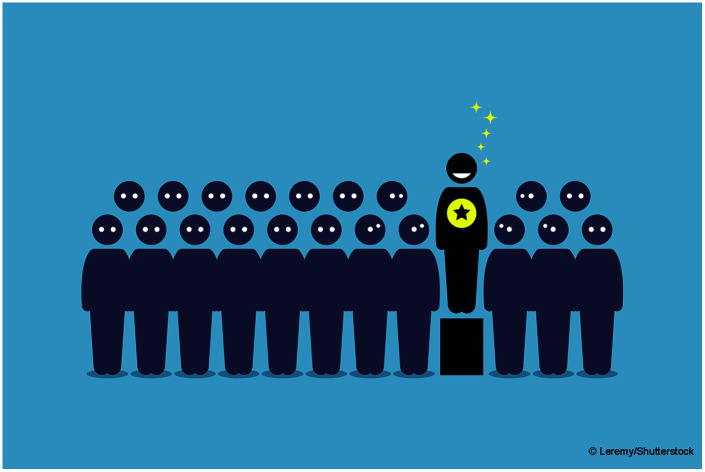

We would like to take this opportunity to thank all of *Chemical Science’s* reviewers, and in particular highlight the Outstanding Reviewers for the journal in 2020, as selected by the editorial team for their significant contribution to *Chemical Science*. We announce our Outstanding Reviewers annually and each receives a certificate to give recognition for their contribution.

We recognize the many and varied contributions our reviewer community make to the high quality of the research published in the journal, so our Outstanding Reviewers from 2020 have been chosen based on a number of different measures.

Our list includes reviewers who provided a much higher than average number of excellent quality reports. However we also recognize that providing a high number of reports is not the only important measure, so we are also highlighting reviewers who provided extraordinarily detailed reports, reviewers who were particularly noted for constructively helping authors to improve their manuscripts, and also reviewers who provided noteworthy and thoughtful adjudicative reports as well.

By widening the criteria for selecting our Outstanding Reviewers from 2020, we are delighted to say this has produced a more diverse and representative sample of our reviewer community.

It is our continuing aim to thank the many people who contribute their time and effort in support of *Chemical Science*. For that reason, later this year we intend to launch our new Reviewer Spotlight feature. There will be more details on this to follow on our website, Twitter and Facebook, however we hope this will be an opportunity for the editorial team to regularly identify reviewers throughout the year, and to further recognize them for their contribution and support.

 

Dr Igor Alabugin

Florida State University

ORCID: 0000-0001-9289-3819

 

Professor Athina Anastasaki

ETH Zurich

ORCID: 0000-0002-6615-1026

 

Professor Louise Berben

UC Davis

ORCID: 0000-0001-6461-1829

 

Professor Holger Braunschweig

University of Wurzburg

ORCID: 0000-0001-9264-1726

 

Professor Shunsuke Chiba

Nanyang Technological University

ORCID: 0000-0003-2039-023X

 

Dr Lillian Chong

University of Pittsburgh

ORCID: 0000-0002-0590-483X

 

Professor R. Graham Cooks

Purdue University

ORCID: 0000-0002-9581-9603

 

Dr Miquel Costas

Institut de Química Computacional i Catàlisi

ORCID: 0000-0001-6326-8299

 

Dr Amitava Das

Indian Institute of Science Education and Research Kolkata

ORCID: 0000-0003-3666-1743

 

Dr Claire Deo

European Molecular Biology Laboratory

ORCID: 0000-0003-0119-3118

 

Professor Paula Diaconescu

UCLA

ORCID: 0000-0003-2732-4155

 

Professor Ryan Gilmour

WWU Münster

ORCID: 0000-0002-3153-6065

 

Professor Frank Glorius

WWU Münster

ORCID: 0000-0002-0648-956X

 

Professor Leticia González

University of Vienna

ORCID: 0000-0001-5112-794X

 

Professor Will Gutekunst

Georgia Institute of Technology

ORCID: 0000-0002-2427-4431

 

Professor Katrina Jolliffe

The University of Sydney

ORCID: 0000-0003-1100-4544

 

Dr Kjell Jorner

University of Toronto and Chalmers University of Technology

ORCID: 0000-0002-4191-6790

 

Professor Mi Hee Lim

KAIST

ORCID: 0000-0003-3377-4996

 

Professor Connie Lu

University of Minnesota

ORCID: 0000-0002-5162-9250

 

Dr Partha Mukherjee

Indian Institute of Science

ORCID: 0000-0001-6891-6697

 

Professor Stephen Newman

University of Ottawa

ORCID: 0000-0003-1949-5069

 

Professor Andrea Rentmeister

WWU Munster

ORCID: 0000-0002-3107-4147

 

Professor Richmond Sarpong

University of California Berkeley

ORCID: 0000-0002-0028-6323

 

Professor Hannah Shafaat

Ohio State University

ORCID: 0000-0003-0793-4650

 

Dr Mattia Silvi

University of Nottingham

ORCID: 0000-0002-0728-7193

 

Dr Zhiyong Tang

National Center for Nanoscience and Nanotechnology

ORCID: 0000-0003-0610-0064

 

Professor Takashi Uemura

The University of Tokyo

ORCID: 0000-0002-1357-3196

 

Professor Andrew Weller

University of York

ORCID: 0000-0003-1646-8081

 

Dr Lin Yuan

Hunan University

ORCID: 0000-0002-1015-5319

 

We would also like to thank the *Chemical Science* Editorial Board and Advisory Board and the chemical science community for their continued support of the journal, as authors, reviewers and readers.

 

May Copsey, Executive Editor

